# Good Nursing

**Published:** 1871-06

**Authors:** 


### GOOD NURSING

Is a thousand fold better than physic for old and young. Many a first-born, whom the mother never fails to mourn as long as life lasts, owes its early death to ignorance and carelessness. The mortality of the British army in the Crimean War diminished one half, with the same medicines and the same physicians, by the aids which Florence Nightingale introduced in the care of the sick. Five years ago, ninety infants in a hundred in the almshouses of Massachusetts died. Some humane women introduced a better system of nursing and care, with the result that now only three of a hundred die. If some of our idle young women, instead of working and waiting to catch some rich man, were to learn how to nurse the sick, they could earn from ten to twenty dollars a week in New York city, all the year round, and which, judiciously invested, would make them independent of the men for life, if they wanted to be ; if they wanted to marry, they could have their pick and choice among a thousand nice young men. What Howard or Peabody or A. T. Stewart will endow an institution for educating young women to be nurses for the sick ? Could there be a nobler right to women than the right which ability gives to nurse the sick and save from death. A high humanity this. But the handsomest must be sent to nurse the men, especially bachelors ; they would be cured in half the time, and would pay double wages.

